# Flavin-Dependent Thymidylate Synthase as a New Antibiotic Target

**DOI:** 10.3390/molecules21050654

**Published:** 2016-05-20

**Authors:** Michael Choi, Kalani Karunaratne, Amnon Kohen

**Affiliations:** Department of Chemistry, The University of Iowa, Iowa City, IA 52242-1727, USA; michael-a-choi@uiowa.edu (M.C.); kalaniudara-karunaratne@uiowa.edu (K.K.)

**Keywords:** flavin, enzyme, mechanism, thymidylate synthase, antibiotic

## Abstract

In humans *de novo* synthesis of 2′-deoxythymidine-5′-monophosphate (dTMP), an essential building block of DNA, utilizes an enzymatic pathway requiring thymidylate synthase (TSase) and dihydrofolate reductase (DHFR). The enzyme flavin-dependent thymidylate synthase (FDTS) represents an alternative enzymatic pathway to synthesize dTMP, which is not present in human cells. A number of pathogenic bacteria, however, depend on this enzyme in lieu of or in conjunction with the analogous human pathway. Thus, inhibitors of this enzyme may serve as antibiotics. Here, we review the similarities and differences of FDTS *vs.* TSase including aspects of their structure and chemical mechanism. In addition, we review current progress in the search for inhibitors of flavin dependent thymidylate synthase as potential novel therapeutics.

## 1. Introduction

**Scheme 1:**
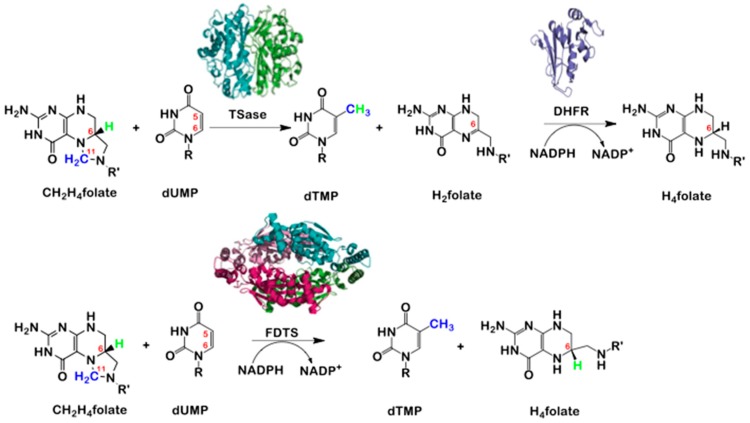
Reactions catalyzed by TSase and DHFR (**top**), and FDTS (**bottom**). Highlighted are the reducing hydrogen in the TSase reaction (green), methylene (blue). R, 2′-deoxyribose-5′- phosphate; R′, (*p*-aminobenzoyl) glutamate; NADP^+^, nicotinamide adenine dinucleotide phosphate; NADPH, reduced form of NADP^+^. To demonstrate that these enzymes are not structurally related, the crystal structure of each protein is presented above its name (PDB IDs: 2KCE, 1RX2, and 1O26, for TSase, DHFR, and FDTS, respectively).

Replication of all cells requires thymidine, a pyrimidine that makes up DNA. Thymidine is synthesized in human cell *de novo*. The last step of the synthesis is catalyzed by the enzyme thymidylate synthase (TSase) coded by the *thyA* gene. TSase catalyzes the conversion of 2′-deoxyuridine-5′-monophosphate (dUMP) to 2′-deoxythymidine-5′-monophosphate (dTMP or thymidylate) using N^5^,N^10^-methylene-5,6,7,8-tetrahydrofolate (MTHF or CH_2_H_4_folate) in a reductive methylation reaction (Scheme 1).

Through this process, dTMP is made, which can then be used in DNA synthesis. Also as a result of the activity of TSase, MTHF is converted to dihydrofolate (DHF or H_2_folate). Since the availability of folate derivatives is a rate-limiting factor in cell replication, DHF must be recycled back into MTHF. The recycling of DHF is catalyzed by the *folA* encoded enzyme dihydrofolate reductase (DHFR), which synthesizes tetrahydrofolate (THF or H_4_folate) (Scheme 1), which is then converted to MTHF by serine hydroxymethyltransferase [1]. The recycling of DHF and the enzymes involved in this cycle are clinically important. Pharmaceuticals which target TSase, such as 5-fluorouracil and raltitrexed [2], are important chemotherapeutics in skin, colon, ovarian, and other cancers [3]. The enzyme DHFR is also targeted by drugs like methotrexate for cancer, autoimmune disorders, and other illnesses [4,5]. *De novo* dTMP production is not only important for humans. The antibiotic trimethoprim is a bacteriostatic inhibitor of DHFR that has both Gram positive and Gram negative coverage [6].

Because of the importance of dTMP to the synthesis of DNA, it was thought that TSase and the enzymes required to regenerate MTHF were essential. However, in 2002, it was reported that a number of organisms did not have these systems in place [7]. Some of those organisms did not have the *Tdk* gene (encoding for thymidine kinase), thus could not even scavenge for thymidine in media or host. 

Instead, they appeared to have a different enzyme, which is encoded by the gene *thyX*. This new enzyme was found to contain flavin adenine dinucleotide (FAD) and use nicotinamide adenine dinucleotide phosphate (NADPH) as the reducing agent. It catalyzes the conversion of dUMP to dTMP, and thus named flavin dependent thymidylate synthase (FDTS). There was also no analog of the MTHF recycling system found in some of these prokaryotes. Altogether it is estimated that approximately 40% of prokaryotes carry the *thyX* gene, though some of these prokaryotes also have the *thyA* and *folA* genes for TSase and DHFR, respectively.

Comparing TSase and DHFR to FDTS shows no sequence or structural homology [8,9,10]. Importantly, a number of human pathogens have FDTS, including *Bacillus anthracis*, *Mycobacterium tuberculosis*, *Helicobacter pylori*, and all *Rickettsia* species (see Table 1) [7,11,12]. The lack of this enzyme in humans and the important role that thymidylate synthesis plays could make FDTS a new antibiotic target. Antibiotic resistance is increasing in tuberculosis and emerging in *H. pylori* and *B. anthracis*, which make the search for new classes of antibiotics more pressing [13,14,15].

## 2. Thymidylate Synthase (TSase) *vs.* Flavin Dependent Thymidylate Synthase (FDTS)

**Figure 1:**
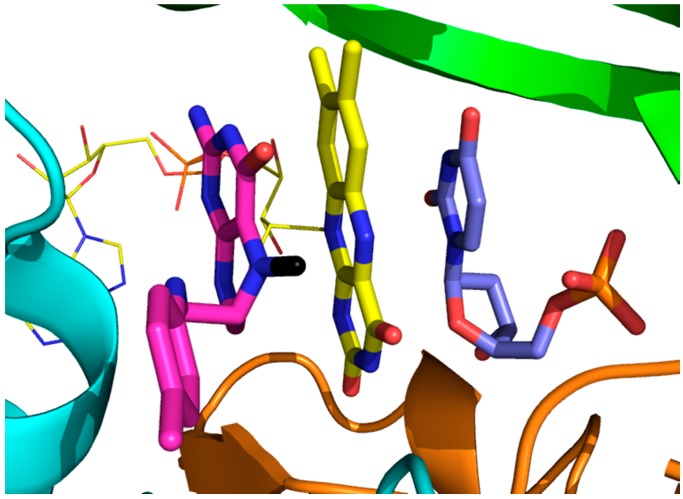
Structure of the active site of FDTS from *T. maritima* with folinic acid (magenta), FAD (yellow), and dUMP (blue) (PDB ID 4GTA) [20]. The three monomers that combine this active site are in green, light blue and orange, and the carbon that is to be transferred is marked in black.

Structurally, TSase is a homodimer (Scheme 1) [18] with one active site in each monomer. FDTS on the other hand is a homotetramer (Scheme 1) [10,19] with four active sites, each one at the interface of three of the monomers (Figure 1). Crystallization of FDTS with different folate derivatives, FAD, and dUMP indicate that the substrates are stacked within the active site (Figure 1). The FAD molecule is sandwiched between the dUMP and folate [19,20]. Since this arrangement places the methylene donor far away from the dUMP acceptor, it suggests an unusual mechanism with respect to the TSase reaction, where methylene is transferred directly from folate to nucleotide.

**Scheme 2:**
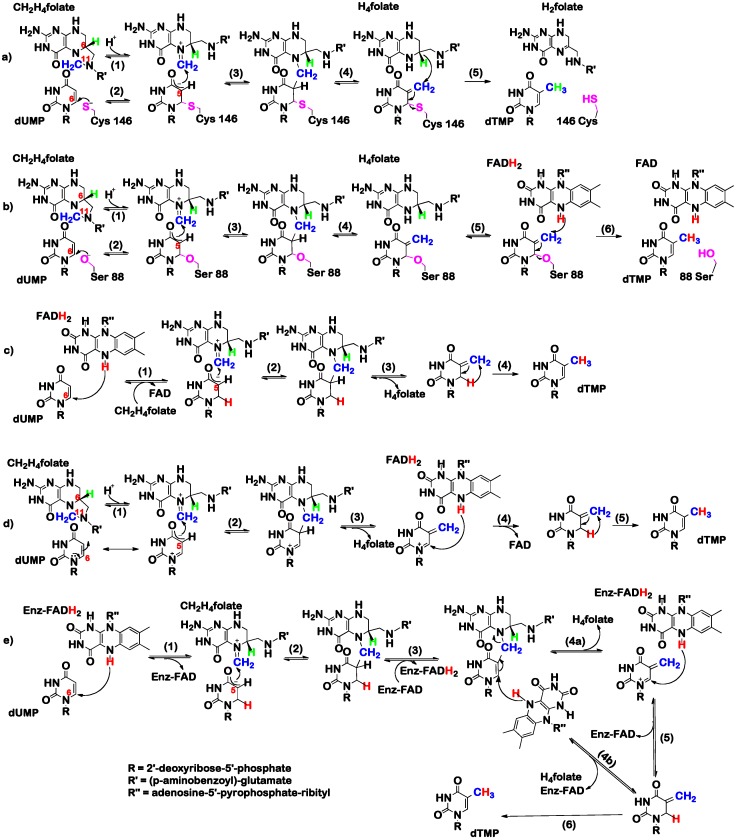
Proposed chemical mechanisms of (**a**) classical TSase; (**b**) FDTS where an active site nucleophile is activating the dUMP ring; (**c**) FDTS where the reduced flavin acting as the nucleophile; (**d**) FDTS mechanism where dUMP is activated by enzymatic polarization rather than Michael addition and (**e**) mechanism of FDTS which combines mechanisms (**c**,**d**) to explain substrate activation by the reduced enzyme. R = 2′-deoxyribose-5′-phosphate, R′ = (*p*-aminobenzoyl)-glutamate, R″ = adenosine-5′-pyrophosphate-ribityl [1,9,22,25,26].

Because of the cycling of the flavin cofactor between reduced and oxidized states, both the oxidative and reductive half reactions should be addressed. NADPH and other reducing agents can be used by FDTS for reducing equivalents during the reductive half-reaction [16]. Formation of the product dTMP occurs during the oxidative half reaction, and following the oxidation state of the flavin spectroscopically indicates that, it gets oxidized during the formation of dTMP [21,22]. Like with TSase, ^14^C radiolabeling studies done on the methylene carbon of MTHF (C11, Scheme 2) demonstrate the incorporation of ^14^C into the dTMP product. However, unlike classical TSase, FDTS does not rely on MTHF for the reducing hydride equivalent, thus no DHF is produced, and no DHFR is needed in FDTS dependent organisms. Studies carried out with [6-3H]-MTHF did not show any tritium incorporation into the dTMP product; this is an evidence against FDTS serving as a bifunctional enzyme with both TSase and DHFR activities [9]. This observation together with the structural differences and the presence of FAD suggested that TSase and FDTS may have different catalytic mechanisms, which prompted mechanistic studies to compare these two enzymes.

The mechanism of TSase (Scheme 2a) has been extensively studied [1,23,24]. In this mechanism dUMP is activated by an enzymatic nucleophile cysteine via Michael addition to the C6 of the dUMP (steps 1 and 2). The methylene is subsequently transferred to the C5 position of the covalently-activated dUMP (steps 3 and 4) followed by reduction (step 5), yielding DHF and dTMP.

In the TSase reaction MTHF provides both the methylene group as well as the reducing hydride to synthesize dTMP [1]. The chemotherapeutic 5-fluorouracil is a mechanism based inhibitor. In the cell it is metabolized to 5-fluoro dUMP, which reacts similarly as the enzyme’s native substrate, dUMP, by being attacked by the nucleophilic enzyme side-chain, but the fluorine on the C5 position is unable to leave (step 4, Scheme 2a) resulting in a dead-end inhibitor for the enzyme [3].

Because the first chemical modification of dUMP in the classical TSase mechanism involves a Michael addition with an enzymatic nucleophile, early-proposed mechanisms of FDTS included an analogous scheme (Scheme 2b). FDTS does not have a conserved cysteine residue within its active site like TSase; instead, a conserved serine residue was proposed as the Michael nucleophile. However, when that serine was mutated to alanine, *in vitro* assays showed that the enzyme was still active [19]. The observation that FDTS does not use an enzymatic nucleophile further suggested a different mechanism from that of TSase.

Furthermore, it was observed that when the FDTS reaction was conducted in D_2_O at low temperature, 60% of the product, dTMP, had deuterium at position C6 [19]. Since in D_2_O the reduced flavin is deuterated, it was proposed that the nucleophile activating the aromatic ring of dUMP by Michael addition might be the hydride from the FADH_2_ (Scheme 2c). In that proposed mechanism, first, the hydride from the reduced flavin does a Michael attack on C6 of dUMP followed by the methylene transfer to the C5 position. Finally, either a 1,3-hydride shift or proton addition-elimination mechanism is needed to yield dTMP. The possibility of N5 of the flavin acting as a nucleophile for the Michael addition at C6 was refuted because it was reported that FDTS reconstituted with 5-deazaflavin was active [19]. This is because the analogous carbon position in 5-deazaflavin cannot act as a nucleophile. However, spectrophotometric experiments following the redox state of the flavin along with radiolabeling have demonstrated that the flavin oxidation occurs late in the reaction [21,22]. This observation lead to another proposed mechanism, one that involves no Michael addition at all. Instead, dUMP is activated by polarization (Scheme 2d) [20,22,26]. In such mechanism, the accumulated negative charge on C5 of the polarized dUMP nucleophilically attacks the Schiff base (step 2 in Scheme 2d) followed by deprotonation and elimination of the THF. The exocyclic methylene dUMP is then reduced by the flavin (step 4) and undergo a 1,3-hydride shift to form dTMP (step 5) [22].

In order to further understand the mechanism of FDTS, intermediates of the oxidative half reaction (where flavin is oxidized and dUMP is converted to dTMP) were trapped under single-turnover conditions. FDTS single turnover experiments quenched by acid showed the accumulation of 7-hydroxymethyl-dUMP which is generated from water addition after the methylene transfer to C5 of dUMP [21]. Although only one intermediate was trapped, the broad peak of accumulation of the acid-quenched intermediate suggested that more than one intermediate was trapped in acid as 7-hydroxymethyl-dUMP [26]. Independent experiments have also suggested two intermediates due to the observation of a spectral flavin change as the reaction progresses followed by the transient disappearance of reactant and product nucleotides [22].

Subsequent experiments [26] demonstrated that incubation of dUMP with reduced FDTS in D_2_O exchanges the hydrogen at the C5 position. This does not occur with oxidized FDTS. Also, when the reaction is done in D_2_O, dTMP becomes monodeuterated in the C6 or C7 position, but the acid-trapped intermediate was found to have no deuterium. A new mechanism (Scheme 2e) was proposed to reconcile this new data. Hydride transfer from the N5 of the reduced flavin occurs at the C6 position prior to methylene transfer to C5. It is then proposed that elimination occurs in step 3 by FAD. Steps 4 through 6 describe hydride transfer and elimination of H_4_folate either through a step-wise or concerted mechanism followed by a 1,3 hydride shift to yield dTMP [26]. Mechanism 2e also accords with the stopped-flow experiment where flavin oxidation happen close to dTMP formation rather than early in the reaction [21,22].

## 3. The Latest Proposed Mechanism for FDTS (April 2016)

**Scheme 3:**
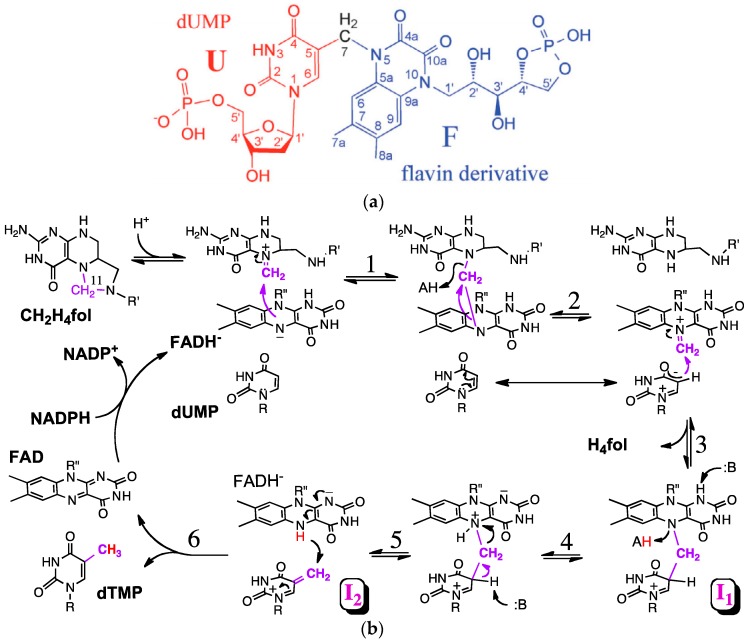
(**a**) Trapped intermediate derivative from quench-flow experiments of FDTS with base identified through NMR, MS, and labeling experiments. The derivative is composed of dUMP (U), a methylene from MTHF (7) and a flavin derivative (F); (**b**) New proposed mechanism of FDTS (see text). I_1_ is the intermediate before base-induced breakdown of the isoalloxazine ring, and I_2_ is the intermediate that is reduced to dTMP upon quenching with base. Both I_1_ and I_2_ undergo water addition under acidic conditions to form 7-hydroxymethyl-dUMP. Modified from reference [17].

Recently, new evidence regarding the mechanism of FDTS was acquired through quenching the reaction in base instead of acid, which yielded a different intermediate. Mass spectrometry showed the base-trapped intermediate to be [M − H]^−^ 705.1212 Dalton. Isotopic labeling and NMR studies elucidated the structure to be dUMP with a methylene bridge to a derivative of the isoalloxazine ring from the FAD (Scheme 3a). The trapping of the labeled methylene carbon from methylene tetrahydrofolate suggests that the isoalloxazine ring of the flavin may play a part as a methylene relay system from the MTHF to the dUMP.

Given the finding that the isoalloxazine ring appears to act as a methylene relay system, and based on the structure presented in Scheme 3a, a new mechanism has been proposed (Scheme 3b). This new mechanism involves an initial Schiff base formation of the MTHF followed by methylene transfer to the N5 of the flavin (steps 1 and 2). Polarization of the dUMP substrate and transfer of the methylene group to C5 of the dUMP leads to a bridged methylene intermediate, I_1_ (steps 2 and 3). A derivative of this intermediate is the bridged intermediate isolated from base quenching of FDTS (Scheme 3a). Methylene transfer to dUMP (steps 4 and 5) followed by flavin oxidation and I_2_ reduction to dTMP (step 6) and oxidized flavin. NADPH then reduces the flavin during the reductive half reactions, and the cycle continues. The relay of the methylene group from folate to flavin and ultimately to the dUMP accords well with the crystal structure where the flavin cofactor is between folate and dUMP (Scheme 3b) [17,20]. Why was this mechanism not proposed in 2012 when the structure was solved (Figure 1). Because of the report that FDTS with FAD substituted for 5-deaza-FAD is still active, and thus N5 of FAD cannot serve in the role proposed in Scheme 3b (step 1). Extensive recent efforts [17] found that actually FDTS with 5-deaza-FAD is not active, and the residual activity reported in [19] was from residual FAD left in the protein. Step 6 indicates reduction of I_2_ at C6, to non-productive isomer if dTMP. This principal mechanism while not very detailed, is in accordance with all current observations, but one: the deuterium found at C6 of dTMP at low temperature [19]. That observation, however, might result from reversible but non-productive reduction of I_2_ at C6, followed by the productive irreversible reduction at C7, leading to the product dTMP.

In the reductive half-reaction of the enzyme, NADPH reacts with the bound FAD of the enzyme to reduce the cofactor. The initial binding of dUMP to the enzyme greatly enhances the rate of the reduction while the presence of MTHF [27] or its analogue, folinic acid [20], reduces the rate of the reductive half reaction, indicating an overlapping binding site of NADPH and folates. This also suggests an ordered binding mechanism where the enzyme first binds to dUMP, the NADPH reduces the flavin, followed by NADP^+^ leaving with the MTHF binding in the vacant site.

## 4. FDTS Specific Inhibitors

Many inhibitors of TSase are in laboratory and clinical use, but specific and efficient inhibitors of FDTS (*K*_i_ at the low nM range) are not yet reported nor in clinical use. Attractive classes of antibiotic drugs against FDTS dependent organisms need to exhibit high specificity toward FDTS and low specificity toward human TSase in order to reduce toxicity. High throughput screening studies and synthesis of inhibitors based on substrate scaffolds have been attempted, and some of these inhibitors achieve an IC_50_ in the 100 nM range against FDTS (Table 2) [28,29,30,31]. 

Encouragingly, lipophilic derivatives of compound **1** in Table 2 have been shown to be inhibitory to the growth of *Mycobacterium* species as well as herpes viruses, and its inhibition of classical TSase was much weaker than for FDTS [30]. Compound **3**’s inhibitory activity against FDTS is reversed by excess dUMP, but compound **4**’s is not [31]. However, for many of the proposed inhibitors, the specificity towards FDTS over TSase, the efficacy of these compounds against bacterial pathogens, and the toxicity towards human cells remain to be further tested. Possible inhibitors to investigate in the future may include folate based molecules that take into account the nucleophilicity of the flavin cofactor towards the methylene of MTHF to covalently trap its N5. For example, electrophilic substituents like epoxides at N5 of folate may be able to create dead-end folate-flavin adducts [17]. An alternative strategy may involve a similarly nucleophilic “warhead” on C5 of dUMP, but those studies are still in their infancy.

## 5. Conclusions

Mechanistic and structural studies of FDTS have demonstrated a catalytic path that is very different from that of classical TSase. Most notably, the use of flavin as the reducing equivalent and as relay system for both the electrons and methylene, and the lack of an enzymatic nucleophile makes the chemistry of FDTS dramatically different from human TSase. The fact that several human pathogens depend on FDTS and the differences between its mechanism to that of human TSase and DHFR, makes it an excellent target for new classes of antibiotics. Pathogens depending on FDTS include organisms that have developed antibiotic resistance, such as multi-drug-resistance tuberculosis (MDR TB). The unprecedented mechanism of FDTS gives hope that specific mechanism-based inhibitors might serve as leads toward synthesis of non-toxic antibiotic drugs. Designing such inhibitors require extensive work in both computational and experimental fields. Additional studies on the mechanistic details of FDTS will provide a basis for the rational design of mechanism-based-inhibitors that may serve as leads to a new class of antibiotics. Finally, the unique chemistry that has so far been elucidated will also provide insight into alternative methyltransferase mechanisms as well as understanding the reactivity of flavins.

## Figures and Tables

**Table 1:** Table of pathogenic bacterial species containing ThyX as well as whether thymidine kinase (Tdk) and TSase (ThyA) or DHFR (FolA) are present [16,17].

Bacterial Species	Associated Disease (s)	Tdk	ThyA	FolA
***Bacillus anthracis***	anthrax	+	+	+
***Borrelia burgdorferi***	Lyme disease	+	−	−
***Campylobacter jejuni***	diarrhea	−	−	−
***Chlamydia trachomatis***	trachoma	−	−	+
***Chlamydia pneumoniae***	pneumonia	−	−	+
***Clostridium botulinum***	botulism	+	+	+
***Helicobacter pylori***	stomach ulcer, gastric cancer	−	−	−
***Mycobacterium leprae***	leprosy	−	+	+
***Mycobacterium tuberculosis***	tuberculosis	−	+	+
***Rickettsia prowazeki***	typhus	−	−	−
***Rickettsia rickettsii***	spotted fever	−	−	−
***Treponema pallidum***	syphilis	−	−	−

**Table 2:** Current inhibitors and their IC_50_ against FDTS and TSase [28,29,31].

Entry	Structure	ThyX IC_50_ (µM)	ThyA IC_50_ (µM)
1	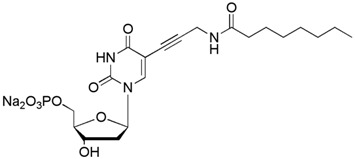	0.91	>50
2	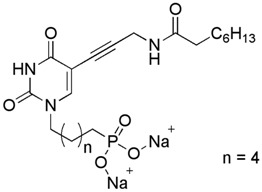	43% inhibition at 50 µM	
3	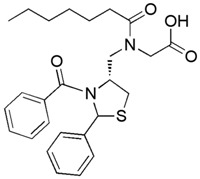	0.13	
4	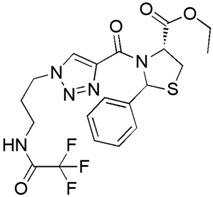	0.057	

## Abbreviations

The following abbreviations are used in this manuscript:

dTMP	2′-deoxythymidine-5′-monophosphate
dUMP	2′-deoxyuridine-5′-monophosphate
TSase	Thymidylate synthase
DHFR	Dihydrofolate reductase
FDTS	Flavin dependent thymidylate synthase
MTHF, CH_2_H_4_folate	N^5^,N^10^-methylene-5,6,7,8,-tetrahydrofolate
DHF, H_2_folate	Dihydrofolate
THF, H_4_folate	Tetrahydrofolate
FAD	Flavin adenine dinucleotide
NADPH	Nicotinamide adenine dinucleotide phosphate
